# Availability Improvement of Layer 2 Seamless Networks Using OpenFlow

**DOI:** 10.1155/2015/283165

**Published:** 2015-02-10

**Authors:** Elias Molina, Eduardo Jacob, Jon Matias, Naiara Moreira, Armando Astarloa

**Affiliations:** Department of Communications Engineering, University of the Basque Country UPV/EHU, Alameda de Urquijo, s/n, 48013 Bilbao, Spain

## Abstract

The network robustness and reliability are strongly influenced by the implementation of redundancy and its ability of reacting to changes. In situations where packet loss or maximum latency requirements are critical, replication of resources and information may become the optimal technique. To this end, the IEC 62439-3 Parallel Redundancy Protocol (PRP) provides seamless recovery in layer 2 networks by delegating the redundancy management to the end-nodes. In this paper, we present a combination of the Software-Defined Networking (SDN) approach and PRP topologies to establish a higher level of redundancy and thereby, through several active paths provisioned via the OpenFlow protocol, the global reliability is increased, as well as data flows are managed efficiently. Hence, the experiments with multiple failure scenarios, which have been run over the Mininet network emulator, show the improvement in the availability and responsiveness over other traditional technologies based on a single active path.

## 1. Introduction

The design of a network requires a robustness study, which is closely related to the use of techniques that minimize service downtime, frame losses, delay, jitter, and, in general, network vulnerabilities that jeopardize the stability of systems. As a rule, network reliability and resilience are improved avoiding single points of failure, and to this end redundancy is one of the widely used methods for preventing the disruption of the normal operation of the infrastructure.

Consequently, the network reliability improvement may enable new applications; especially those critical use cases that require minimum latency and loss of information. In this sense, we highlight the following environments.

- Automation systems:
  - *Smart Grid*. The utility industry and substation automation applications have to accomplish the critical mission of providing power supply in transmission and distribution grids. In accordance with the* Guidelines for Smart Grid Cyber Security* [1] issued by the National Institute of Standards and Technology (NIST): “although the time latency associated with availability can vary, it is generally considered the most critical security requirement.”
  - *Industrial Control*. It is present in factory automation, process industry and motion control.
- Transportation: reliable solutions are being implemented in different sectors, such as traffic control systems, vehicular networks, or avionics.
- Audio/video: transmission of events whose streaming requires low latency without frame losses.
- Data center: these infrastructures are inherently redundant, establishing multiple paths between hosts.
- Access and transport networks: they include mechanisms of protection to maintain the performance, minimizing service interruptions and fulfilling Service Level Agreements (SLAs).

In particular, we focus on the reliability of Ethernet Local Area Networks (LANs) and present an overview of layer 2 redundancy protocols, showing the active redundancy approach as a necessary strategy to provide zero recovery time. The fact is that, despite new insights have expanded the Ethernet standards to be redundant, there are not many options to provide zero loss performance in LANs; among them we focus on a solution recently standardized by the International Electrotechnical Commission (IEC): the Parallel Redundancy Protocol (PRP) that, based on the duplication of data and resources, enables a seamless communication in single-failure scenarios.

In this study, instead of using PRP in conjunction with common spanning tree technologies, we propose to combine the OpenFlow and PRP protocols for implementing a further active redundancy, achieving zero recovery time in case of multiple simultaneous failures; for which we rely on the capabilities of PRP nodes, along with flow-oriented control and flexibility features of OpenFlow. Moreover, taking into account that resilience is closely related to the network dynamicity, we describe the potential of the Software-Defined Networking (SDN) paradigm, in which “the control and data planes are decoupled, network intelligence and state are logically centralized, and the underlying network is abstracted from the applications” [2], to achieve a better utilization of available resources in situations of active redundancy for facilitating its management and increasing responsiveness, also considering the challenges of a centralized approach.

In summary, the aim of this proposal is to ensure a high availability while improving the efficiency and effectiveness of PRP networks, which may serve as an enabling technology for the development of, for example, emerging industrial wireless networks based on PRP solutions [3].

The rest of this paper is organized as follows.  Section 2 analyzes the relationship between redundancy and availability, and some traditional layer 2 redundancy protocols; Section 3 provides an overview of the IEC 62439-3 specification, outlining the capabilities of PRP; Section 4 describes how the SDN paradigm may be incorporated in redundant networks and presents our proposal, whose results are shown in Section 5; Section 6 contains related and future work. Finally, in Section 7 we present the conclusions.

## 2. Redundancy and Availability

In this section, we outline different redundancy methods in connection with the availability of resources. Subsequently, several layer 2 redundancy techniques are summarized.

### 2.1. Types of Redundancy

Redundancy takes two forms: temporal and spatial, while the first form replicates the information over time in a distributed manner, in the spatial redundancy the components or data in a network are replicated, which is the object of study. Conventionally, two types of redundancy are distinguished.

**Figure 1:**
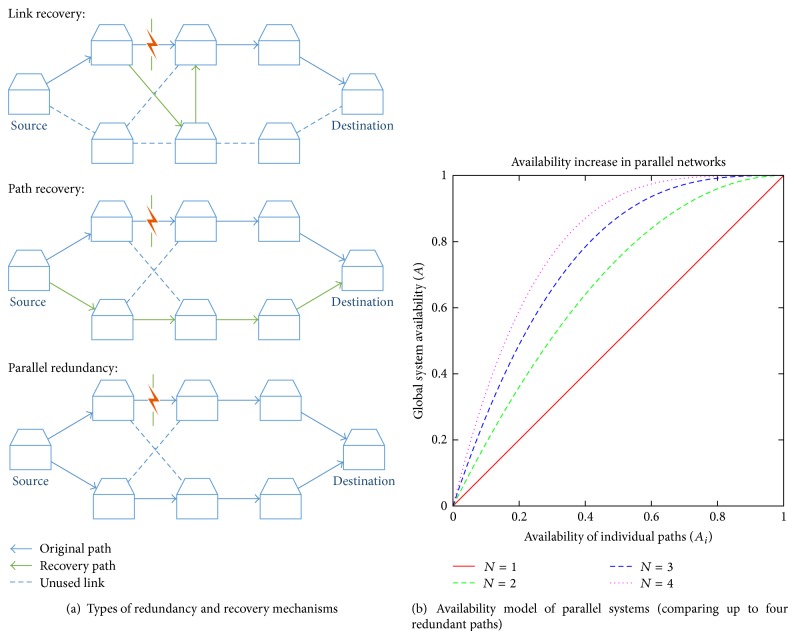
Relationship between redundancy and availability.

- Standby redundancy: through passive resources, these redundant networks switch from an active to a secondary network connection. We can distinguish between partial, which only overcomes the failed link/node, and global recovery, where the whole path is reconfigured (Figure 1(a) exemplifies these principles). In any case, two different schemes can be considered:
  - Protection is schemes where the standby paths are precomputed in a proactive way.
  - Restoration is mechanisms that define recovery network elements reactively in the face of failures and changes in the network.
- Both approaches result in a certain communication downtime, but the protection typically incurs in a lower recovery delay than the restoration approach.
- Active or parallel redundancy: multiple copies of the same data are transmitted along multiple paths simultaneously. The routes can be link-disjoint or node-disjoint for tolerance to link and node failures, respectively. The receiver expects incoming traffic on different routes, so it always receives the information transmitted as long as all paths do not fail simultaneously. This approach eliminates any downtime and ensures that no data are lost due to a single failure.

As can be drawn from the different redundant mechanisms, one of the main differences lies in the switchover time, in which time can be divided into

1. Detection time based on monitoring the communication paths in order to detect failures. Common to all types.
2. Provision time: if any failure is detected, the network control plane must calculate an alternative path. It only affects the restoration case.
3. Switching time to the alternative path and the subsequent communication reestablishment. It generally does not influence in the case of parallel redundancy.

In the design of a network, different redundant methods must be determined on a risk versus reward tradeoff, assessing the need to reduce recovery times and the number of redundant paths compared to other factors, such as management and deployment costs. Obviously, the use of concurrent paths also implies an increase of resources and parallelism management, so they are oriented toward critical use cases as mentioned in Section 1.

### 2.2. Availability Calculation

The availability of the communication connection is essential, however it never can be totally guaranteed. It is defined in ITU-T Recommendation E.800 as follows: “availability of an item to be in a state to perform a required function at a given instant of time or at any instant of time within a given time interval, assuming that the external resources, if required, are provided”; whereas ITU-T Recommendation Y.1563 assesses performance parameters for the specific case of Ethernet service availability. In [4], the authors illustrate an exhaustive analysis of network availability and different recovery methods, applied to several technologies.

From a general view, the availability of the network (*A*) can be quantitatively defined by the parameters known as Mean Time Between Failures (MTBF) and Mean Time To Repair (MTTR) with the expression (1). As can be understood, resilience and redundancy are closely related through fault detection and isolation techniques, which are covered by the Operation, Administration and Management (OAM) tools. Depending on the fault nature, the failover period may be reduced and, therefore, the availability of a network is improved. This may be achieved by automating the prevention and detection of certain faults, while on occasions, the operators diagnosis and decision-making will be totally necessary. Consider

(1) $$\begin{array}{c} A=\frac{\text{MTBF}}{\text{MTBF}+\text{MTTR}}. \end{array}$$

Through an availability model based on Reliability Block Diagram (RBD), we can crudely estimate the impact of parallel redundancy on the overall network availability. Because the availability of a single path (*A*_*i*_) is defined by summation of the individual availabilities of the network equipments and transmission links, the availability of parallel systems (*A*_*p*_) may be calculated per the equation below:

(2) $$\begin{array}{c} A_{p}=1-\overset{N}{\underset{1}{\prod }}\left(1-A_{i}\right). \end{array}$$

The network availability is improved in accordance with redundancy scheme, as shown in Figure 1(b), where up to four parallel paths are compared if we assume that the availability of each connection path is the same.

### 2.3. Traditional Layer 2 Redundancy Technologies

Here we list some of the most relevant approaches to provide redundancy in layer 2 networks. A more extensive analysis can be found in [5].

#### 2.3.1. Spanning Tree Approach

Given that in IEEE 802.3 Ethernet standards there is no mechanism to discard duplicate frames or time-to-live field, loops should be avoided. To that end, the more widely used protocols are based on the spanning tree approach that, employing a distributed algorithm, disables redundant network links for obtaining loop-free topologies. In a failure event, one or more disabled links are reactivated. Under this umbrella, we highlight some standardized protocols, such as the Rapid Spanning Tree Protocol (RSTP, IEEE 802.1D), which upgrades the original STP standard, and Multiple Spanning Tree Protocol (MSTP, IEEE 802.1s). In any case, the resulting topologies do not take advantage of all physical redundant links and, therefore, data do not necessarily follow the shortest path, without achieving the optimal delay, which is considered useful for time-sensitive services. Moreover, a common shortcoming of these protocols is that they do not guarantee a deterministic failover behavior. For example, the RSTP fault recovery time depends on the configuration parameters and the location of the fault; in [6] a method to calculate the maximum recovery time in a ring configuration is provided.

#### 2.3.2. Link Aggregation

These protocols (e.g., IEEE 802.1ax) can be considered as redundant techniques since they allow us to associate several ports to a single logical interface. This enable load balancing, reducing the failover time.

#### 2.3.3. Shortest-Path Protocols

Recently, several alternatives to Spanning Tree-based protocols have been developed. We highlight Transparent Interconnection of Lots of Links (TRILL, IETF RFC 6325) and IEEE 802.1aq. Both compute shortest paths avoiding loops and do load balancing through multiples paths. However, they are not conceived as active redundancy protocols. In the specific case of TRILL, [7] shows that it results in “an enhanced alternative to RSTP”; however it “is still unable to meet the required convergence time claimed by the Smart Grid requirements.” Additionally, there are recent attempts [8] to build active protection paths in TRILL networks so that “when a link on the primary distribution tree fails, the preinstalled backup forwarding table will be utilized without waiting for the reconvergence, which minimizes the service disruption.”

## 3. IEC 62439-3 Parallel Redundancy Protocol

Ethernet technology is being selected for many critical projects which demand dependable communication infrastructures that meet stringent reliability requirements. Nevertheless, technologies such as those mentioned above may not be valid in terms of recovery times. Therefore, this section presents a representative case in which it is necessary a minimal failover time, describing the IEC 62439-3 PRP as a mechanism for achieving it.

### 3.1. IEC 62439 and IEC 61850

The IEC 62439 standard suite includes a set of redundancy control protocols for industrial automation. These protocols are oriented to support different topologies and recovery times; from which only two options (PRP and High-Availability Seamless Redundancy, HSR), defined in IEC 62439-3 [9], are able to provide bumpless redundancy in case of any single network failure. To achieve this, IEC 62439-3 proposes that the devices be connected by active redundant links; the specific PRP operation mode and the Ethernet frame specification will be described below.

PRP and HSR can be useful in many applications where high availability and low latency are required. But the most important use case is related to its adoption by the IEC 61850 specification, one of the most widely accepted standards for power system communication. This standard defines, inter alia, the requirements to be met by the network responsible for providing the connectivity service in power automation systems, which is based on Ethernet LANs. The IEC 61850 Edition 2 standard (2011) introduces more demanding applications than its first edition (2004), whose maximum transfer time for different messages types are defined in IEC 61850-5, tolerating a maximum recovery time in the order of 4 ms for the most stringent data requirements. For example, [10] focuses on substation automation systems and it studies the performance of time synchronization services in RSTP and PRP networks where multiple network failures are simulated and, obviously, PRP is much more tolerant to such failures.

Additionally, it is necessary to note that although upper layers can include resilient methods responsible for the detection of duplicates and error recovery (e.g., TCP), the achieved recovery time could not be sufficient to provide minimal latencies. Therefore, IEC 61850 introduces different services (defined by IEC 61850-8-1 and IEC 61850-9-2) that, using multicast service, are mapped onto the Ethernet link layer for functions that need to transmit time-critical data.

### 3.2. PRP Operation Process

In PRP, specified by the IEC 62439-3 Clause 4, each device is connected in parallel to two LANs. PRP is fully implemented in the end-nodes, called Double Attached Node (DAN), so that network switches are protocol-agnostic, and even PRP specification is independent of intrinsic redundancy used in the LANs. Consequently, two networks can differ in topology, delay and performance. Only the requirements imposed on the networks “are having no connection between them, as they are assumed to be fail-independent and having an identical MAC-LLC level” [9]. In this sense, a PRP end-device has the same MAC address in both interfaces. Another specific requirement is that the switches have to allow oversized frames, since DANs extend the Ethernet header, meaning that oversized frames (with length of up to 1532 bytes) can take place.

Regarding flexibility and compatibility with off-the-shelf devices (Single Attached Node, SAN), they can be attached directly to one network without having to be aware of PRP. Additionally, the standard specifies how to use PRP proxies, called Redundancy Box (RedBox), to which SANs can be connected redundantly to both networks (denominating them as Virtual DAN).

With respect to the operation process, a DAN implements a Link Redundancy Entity (LRE), responsible for managing the redundancy and duplicates transparently to the upper layers. This is done as follows.

1. When the Entity receives a message from upper layers, it creates two frames by adding the so-called Redundancy Control Trailer (RCT) and calculating a new checksum.
2. The Entity sends out the frames through its both ports at the same time. These two frames traverse the two independent networks.
3. At the destination node, the LRE has two operation modes to handle the received frames.
  - Duplicate Accept (“for testing purpose” [9]) or Duplicate Discard: the latter, which is the most common mode, ensures that the upper layer receives only the first data frame. For this purpose, the LRE must maintain a buffer of the first received frames to recognize and discard duplicates. The buffer implementation affects the algorithm to detect duplicates (not specified by the standard); for instance, decisions about timeouts and buffer sizes must be consistent with network performance goals.
  - In both cases, the LRE removes the RCT and forwards the received frame to its upper layers. In case that the duplicates are not discarded, upper protocols, such as IP and TCP protocols, can tolerate receiving and removing duplicates.

In addition, PRP provides a mechanism for the network supervision, so that each DAN monitors the status of each LAN and other PRP devices. This facilitates the control of network errors, as well as discovering other DAN. To this end, multicast frames, identified by a specific Ethertype (0x88FB), are used.

### 3.3. PRP Ethernet Frame Format

**Figure 2:**
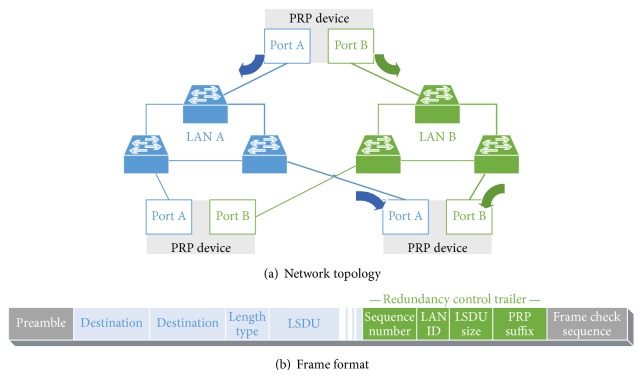
PRP operation process and frames.

Figure 2 shows a schematic PRP diagram and the frame format, since, to enable detection of duplicated frames, PRP nodes add the RCT, 6 bytes long, structured as follows.

- Sequence number (16 bits): the source increments it for each frame sent, allowing a receiver to detect missing messages and permanent failures.
- LAN A/B Labeling (4 bits): it identifies the network to which send the frame. The specification defines only 0xA and 0xB codes.
- LSDU size (12 bits): it indicates the size of the Link Service Data Unit (LSDU) including the RCT.
- PRP suffix (16 bits): it coincides with the Ethertype (0x88FB).

In summary, PRP is a simpler technique and more easily implementable than other approaches such as [11], which proposes a multiple path Ethernet scheme, along with a congestion control and packet retransmission mechanisms in order to be able of transmit data through parallel paths in a reliable manner.

### 3.4. Comparison with HSR

High-Availability Seamless Redundancy (HSR, IEC 62439-3 Clause 5) can be considered a special version of PRP applied to certain topologies. In contrast to PRP, HSR requires only an additional path between two nodes; on that ground, HSR is typically used in ring topologies. Therefore, in an HSR network, the end-nodes are connected with each other, without needing an external intermediary. To this effect, each HSR device incorporates a bridge function that forwards frames from port to port. These differences make each one of them more appropriate in certain use cases, so we summarize the pros and cons of each protocol.

- While the PRP scheme depends on the network elements and supports two independent LANs of any topology, HSR is limited to ring-based topologies. This is very relevant for the development of our approach. Reference [12] describes different robust topologies that employ PRP and HSR jointly.
- One limitation of PRP is that it is not strictly deterministic, since communication delays may vary depending on the topologies. In contrast, HSR facilitates calculating latencies since it is only necessary to know the number of nodes and their corresponding switching time. Although ring topologies also present inherent limitations, such as the maximum number of hops that does not cause the maximum latency is exceeded.
- While PRP means a duplication of network equipment, HSR does not suppose this overhead, making it less expensive to deploy and maintain than PRP.
- The latter implies that HSR works without dedicated Ethernet switches. By contrast, HSR nodes must implement switching function between their two ports. Accordingly, HSR should be implemented in hardware to meet acceptable time requirements; on the contrary, the PRP nodes can implement the LRE in software, which is also important for our purposes (this is not so in the case of RedBoxes).
- These requirements are related to the flexibility to accommodate standard nodes: unlike PRP, SAN cannot be inserted into HSR topologies.

Regarding other IEC 62439 redundancy protocols, a further detailed analysis can be found in [13], where the authors compare the different specifications. Among them, we also highlight the IEC 62439-4 Cross-Network Redundancy Protocol (CRP) and the IEC 62439-5 Beacon Redundancy Protocol (BRP), which do not provide a seamless communication since they implement a standby redundancy but, however, they allow us to establish cross links between parallel LANs, which can be considered a limitation of PRP, which can be overcome easily by using our scheme, as described in Section 4.2.

## 4. Proposed Architecture

The following is a description of the OpenFlow protocol upon which our approach is based, as detailed subsequently.

### 4.1. OpenFlow-Based Control Plane

One of the most significant SDN technologies is OpenFlow, which is promoted, standardized, and supported by the Open Networking Foundation [2]. By means of the OpenFlow protocol, a controller can access and define the switch data path, adding, updating and deleting flow entries in forwarding tables. These tables contain multiple match fields (ingress port, metadata and packet headers), priority and actions associated with each flow entry [14]. The establishment of forwarding rules can be performed in a reactive mode, in which the controller dynamically inserts entries in response to switches requests; or through a proactive controller that prepopulates the flow tables statically, which is required by time-sensitive scenarios.

Although the OpenFlow centralized architecture may pose scalability issues and it seems to contradict with features required in critical time-sensitive environments, since version 1.2, OpenFlow allows switches to set backup multiple controllers or balance the load among them, avoiding single points of failure. Reference [15] studies the use of a distributed control plane and the optimal placement of controllers to achieve a better failure tolerant in Wide Area Networks (WANs). Likewise, article [16] simulates the latencies between switches and a different number and locations of controllers to find the appropriate recovery process after link failures, proposing a robust architecture against disasters.

Additionally, starting on version 1.1, the OpenFlow tables support a fast failover group entry [14] to accelerate the detection and fault recovery by acting directly on the OpenFlow switches without interacting with the controller.

In summary, as concluded in [17], “a centralized control also has advantages regarding network recovery. In a distributed network, recovering from a broken path can be a slow process. However, an OpenFlow controller is network-aware and it can find the new path faster.”

With regard to opportunities, recent projects have studied the use of OpenFlow in redundant topologies. Particularly, we highlight two projects aimed at increasing the resilience where the duplication management function is relegated to the end-nodes, as occurs in PRP:

- OLiMPS [18]: the Multipath TCP (MPTCP) technology enables, among other features, to do load balancing by using multiple paths between an end-to-end connection. Although MPTCP does not support the simultaneous transmission of the same information in different paths, it is notable the shown interest for using OpenFlow in the computation and provision of multiple link disjoint paths. Moreover, this paper studies the architecture reactivity when some links go down.
- OpenRoads [19]: where OpenFlow is used to establish fully active redundancy and, consequently, improving mobility services. It is based on the fact that the nodes are able to maintain a redundant communication by different wireless technologies (WiMAX and WiFi).

However, it is noteworthy that multipath approaches oriented to centralized load balancing are not the focus of this study because, as stated in [20], “in safety critical systems, structural redundancy is typically not used to increase bandwidth, but to send redundant information over redundant paths,” which is the focus of our proposal.

### 4.2. OpenFlow as a Mechanism for Improving PRP Performance

Here we describe an architecture that, based on the awareness of network configuration and traffic load, combines OpenFlow and PRP with the aim of creating multiple paths for pertinent data flows to achieve a better reliability. Therefore, unlike traditional deployments, in this approach the network control plane is not agnostic about PRP nodes.

Specifically, for establishing more than a single path between two PRP nodes, we distinguish two different implementations:

- DAN-based operation mode: in which the new redundant paths are set in one or more LANs. Consequently, a PRP node receives more than once the same frame through each LAN, which is consistent with the IEC 62439-3 standard since the duplicated frames within 400 ms must be handled by the LRE.
  - While a common deployment, where PRP nodes are connected through two spanning tree-based networks, is impaired when a second failure occurs in one LAN during the recovery period of the another one, in our proposal the performance is not degraded (without considering both failures in the node access links).
  - Moreover, this design enables the establishment of cross-links between each path, which is not possible in traditional PRP deployments because, as stated in [21], LAN A and LAN B cannot connect to each other “since both frames have the same MAC address, the switches would constantly change their address table and this might lead to unstable network conditions.” As mentioned before, CRP and BRP provide cross-redundancy, but they do not take advantage of different paths simultaneously.
- SAN-based operation mode: it allows network designers to increase notably the availability without having to deploy a complete redundant system. Hence, in this operation mode, a PRP node is connected to a single LAN, in which an OpenFlow controller configures more than one path for the transmission of the same content. Thus, the PRP device still receives duplicated frames through its only single interface, having to discard duplicates. This new operation mode reduces redundant resources (cabling and hardware) and, therefore, its CAPEX (Capital Expenditure) and OPEX (Operational Expenditure).

In both cases, the OpenFlow controller pushes flow entries that replicate certain unicast traffic along predetermined multiple paths towards the destination. The first mode means an increase in the availability compared to a common PRP deployment based on, for example, RSTP, whereas the latter is not as robust as the first one since nodes are not doubled attached, but it involves an improvement over a traditional layer 2 LAN with non-PRP compliant nodes.

**Figure 3:**
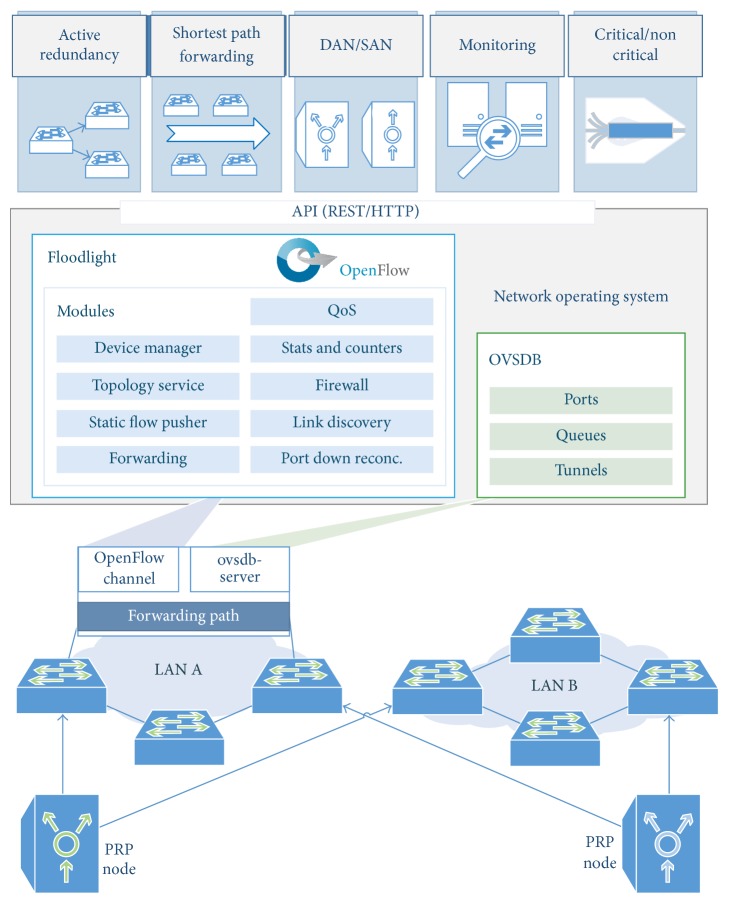
Proposed architecture: NOS and applications.

**Figure 4:**
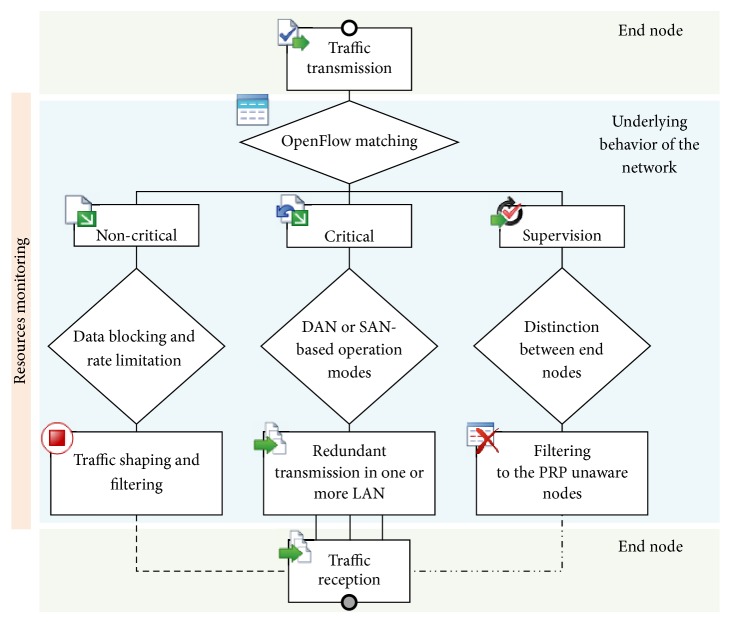
Process flow for identifying and forwarding data traffic.

Regarding the implementation, we have chosen the Floodlight controller [22], which provides a fully functional control plane responsible for tasks such as topology and device discovery, path computing and loops prevention. As shown in Figure 3, the proposed development forms a Network Operating System (NOS) where we can identify different modules that, through a Northbound API based on JSON Representational State Transfer (REST), allow us to design multiple applications running on NOS to take effect on forwarding path of switches. Consequently, through a central interface, the control plane may receive diverse information, such as commands or status alerts, which may be translated into flow entries that are automatically populated in the deployed OpenFlow switches. Then it instantly varies the behavior of a network, being able to allocate resources to different type of traffic, exposing more paths to increase reliability, etcetera.  Figure 4 illustrates the process flow for identifying and forwarding data traffic.

### 4.3. Other Advantages

Although our main objective is to increase the availability, the proposed scheme also improves the network utilization through a network global view and dynamic actions, processing the data path and managing resources efficiently. We have implemented the following functions.

#### 4.3.1. Shortest Path Forwarding

In order to meet timing requirements, latency analysis is a required procedure. Reference [23] studies the components of delay in IEC 61850 networks and it compares the advantages of shortest paths over spanning trees. With our scheme, the fact of using certain OpenFlow controller capabilities represents, by default, an advantage with respect to spanning tree deployments because this allows to compute and set the flow entries that form the shortest path in each LAN. In our case, the Floodlight controller is aware of the network topology through topology discovery services (Figure 3) based on the Link Layer Discovery Protocol (LLDP, IEEE 802.1AB) and we use the Forwarding Floodlight module that is able to perform unicast traffic forwarding along the shortest path in mesh topologies, which is not possible with the Learning Switch module [22].

#### 4.3.2. DAN/SAN Awareness

In spite of the fact that, by default, multicast PRP supervision frames flood the LANs and reach all devices, they are only interpreted by PRP nodes. Although “a RedBox should be configured to stop the transfer of the supervision frames to the SAN devices, so there is no supervision frame flooding to the SANs” [24], this is not required for switches to which the SAN nodes are directly connected. Consequently, this traffic is received by them, in addition to loading the network needlessly. For the purpose of reducing such traffic, we propose a supervision frames filtering that uses the Device Manager and Firewall Floodlight modules (Figure 3); so that, periodically, the control plane learns about devices, taking information about MAC and IP addresses, as well as their attachment points to networks, being aware of nodes that are SAN or DAN. Accordingly, it denies the mentioned multicast frames at egress ports/switches. Therefore, the platform ensures that the supervision flows, which are determined by the Ethertype (0x88FB) and a unique multicast address in the same network, only reaches devices with two interfaces; minimizing the amount of global traffic.

#### 4.3.3. Critical and Noncritical Traffic

PRP compliant devices duplicate all packets regardless of their priorities, which entails that the available network bandwidth is halved. However, this may be inefficient to meet requirements of the applications in many aspects, such as scalability. Consequently, it could be interesting to filter noncritical traffic in order to free resources. For this purpose, the implementation is able to filter TCP/IP traffic, which can be performed in two different modes.

- Data blocking: it prevents the noncritical traffic propagation in one of the LANs, which is performed by using Firewall module (Figure 3). Therefore, this is in accordance with the suggested in [20]: “while critical messages are sent in both directions, it is sufficient for noncritical messages to be sent in one direction only.”
- Data rate limitation: our platform allows network designers to distinguish flow types by establishing traffic shaping policies in a centralized way. In particular, we use the Quality of Service (QoS) module, published in [25], that makes possible to push specific traffic to different queues (OpenFlow supports QoS [14] by setting the network Type of Service (TOS) bits and enqueuing packet), which must be previously created and configured on the particular switch. In our case, because the implementation is performed with Open vSwitch (software switch that supports OpenFlow [26]), the resources are provisioned via the Open vSwitch Database Management Protocol (OVSDB, standardized in [27]) with* ovsdb-client* and* ovsdb-server*. As a result, OpenFlow actions are populated together with QoS policies, take into consideration priority requirements.

#### 4.3.4. Responsiveness and Resources Monitoring

Continuous passive monitoring of network elements, and its integration into the control plane, allows the controller to act dynamically. Among the tools for traffic passive monitoring, we use the OpenFlow protocol itself, which allows the controller to retrieve counters per flows, ports and queues from controlled switches [14]. For example, paper [28] uses this feature for implementing network monitoring tasks and to estimate a traffic matrix, namely the traffic volume from every ingress point to every egress point, for traffic engineering purposes.

Our development is aware of the status of network resources, such as the throughput, detecting network stress events during which delays may increase. In particular, the proposed scheme makes use of flow statistics to adapt the network to the instantaneous needs, including the following actions.

- Enabling and disabling redundant paths according to the resource utilization, thereby, the platform allows us to mark thresholds for triggering different OpenFlow actions; specifically, a monitoring application receive per-flow meters via the Floodlight REST API and when a threshold is exceeded, the multiple paths are flushed of redundant data and vice versa.
- Supervision frame rate checking which is possible because the monitoring application retrieves counters about multicast frames and it checks that they are in line with the expected rates.

On the other hand, disasters and large-scale events may cause multiple points of failure, of which is difficult to determine a complete knowledge in situations where the redundancy control protocol is delegated to the end-nodes, such as defined by IEC 62439-3. As we described previously, PRP allows to control the network integrity and to detect errors. Nevertheless, the provided mechanisms only are measured in a counter, which is typically accessible via the Simple Network Management Protocol (SNMP, IETF RFC 1157). This methodology may be complemented by using OpenFlow, which can greatly improve the response performance in disasters, with the global view provided by the controller. This involves the integration of disaster management systems with the control plane, identifying nonaffected routes and where the resource management system must take into account not only the availability of resources and policy, but also the QoS requirements of the application. This approach is clearly reflected in [29] where different disaster metrics are detected, evaluated and corrected by an OpenFlow controller.

## 5. Performance Validation

In this section we present the emulation tests and results that serve to evaluate the capabilities of the proposal.

### 5.1. Emulation Conditions

In order to build OpenFlow scenarios, the Floodlight controller interacts with Mininet [30], the most widespread tool for emulating SDN-based networks, that “creates virtual networks, running real kernel, on a single machine.” Additionally, with the aim of sending and receiving redundant frames, we use the* PRP Stack* software, published in [31], so that each emulated host over Mininet supports PRP, and therefore, it is able to detect and discard duplicate frames, as well as send supervision multicast messages. Thus, each one has two network interfaces which are virtualized in a single PRP interface. These devices send a PRP supervision message every two seconds.

The results presented here correspond to an out-of-band control-plane configuration in which Mininet uses Open vSwitch to create a set of Ethernet bridges that communicate with Floodlight, which runs on another machine, through independent network resources; although Open vSwitch allows in-band configurations, where data and control planes share the same resources. Thus, failures that affect OpenFlow traffic are excluded from the analysis. For these situations, in [32] the authors implement restoration and protection mechanisms in in-band OpenFlow networks and show recovery times for data and control traffic.

Regarding controller redundancy, both Open vSwitch and Floodlight allow backup configurations, in which bridges communicate preferentially with the master controller, otherwise with the slave one. Moreover, the Open vSwitch has a fall-back mode in case of controllers falling, when it changes to standalone mode (without external controllers).

With regard to the emulation parameters, Mininet uses* NetEm* tool [33] for emulating various capabilities of links, this allows us to set different network conditions, such as

- Frame loss rate:* NetEm* allows to emulate variable packet losses. The smallest possible nonzero value causes 1 out of 43103448 packets to be randomly dropped [33]. In addition, we have modifed Mininet to enable decimal values of rate inputs.
- Mininet emulated links use* Linux Traffic Control* to configure packet queues on interfaces, and it is possible to fix their bandwidth, which serves to characterize saturations and path performance degradation. In our case, the emulated nodes are attached by 100 Mb/s Ethernet links and switches are interconnected by 1 Gb/s links in a ring topology.
- We have not included any additional synthetic delay despite being possible in* NetEm*.

Regarding traffic flows, we use the* iperf* tool for generating UDP flows, which serves to obtain the recovery connection time; since, as defined in [34], test tools that offer monitoring the number of lost frames allow us to know the recovery time of a system (*R*), calculated by the following expression:

(3) $$\begin{array}{c} R=\frac{\text{Lost}\;\text{frames}}{\text{Offered}\;\text{rate}}. \end{array}$$

In all experiments,* iperf* was configured to send packets at a rate of 50 Mb/s. As a result, a device will be continuously sending packets to determine the packet loss rate when network elements fail. Consequently, this development allows us to compare the improvement in recovery time and packet loss rate between different technologies.

### 5.2. Recovery Time Comparison of STP, RSTP and OpenFlow with OVS

The recovery time is an essential parameter to evaluate the quality of a network. Firstly, taking into account that active redundant networks provide seamless communication globally if one switch or link fails, it is relevant to obtain a comparative among previously mentioned technologies, especially STP, RSTP, and OpenFlow. Because current version of Open vSwitch is not compliant with RSTP, we have employed a recent implementation (*ovs-rstp* [35]) that does support it, from which there are no references about the improvement in the reconfiguration time.

As mentioned before, the proposed emulation platform allows us to switch off a network element, either nodes or links. In the shown test, one of the deployed bridges is disabled and the network acts depending on its configuration. For the specific case of OpenFlow, we use the following components.

- When a bridge goes down, automatically Floodlight receives a PORT_DOWN notification (Port Status message [14]) from Open vSwitch.
- Given that the source sends data continuously, the forwarding tables of Open vSwitch maintains the previous flow entries as matchable (data reception period is smaller than the table entry timeout). In our case, to avoid this behavior, the platform loads the Port Down Reconciliation module that, based on LLDP, is responsible for “reconciling flows across a network when a port or link fails” [22]. Regarding the controller response time, it is necessary to emphasize that it is not delayed by other requests, and the mean Round-Trip Delay Time (RTT) between the Mininet machine and the Floodlight host is only 0.3596 ms, measured by* ping* tool.
- Afterwards, the proper shortest path is recomputed and the network retains connectivity between nodes and the succeeded traffic flow can be verified.

**Figure 5:**
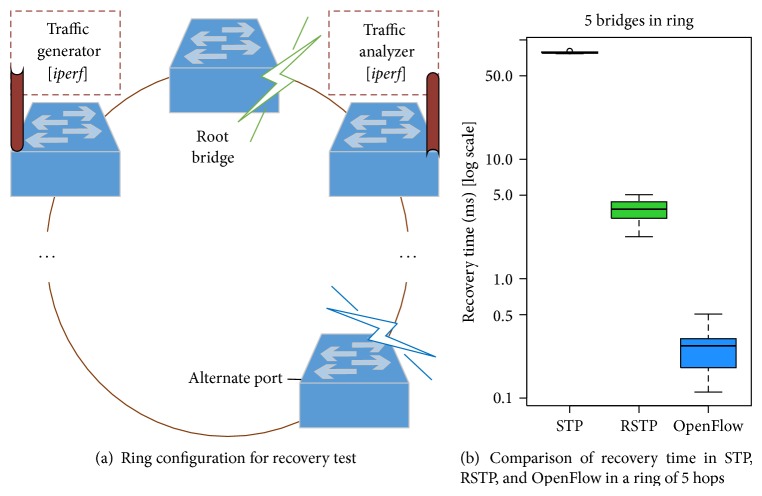
Response time test.

Figure 5 addresses how the different technologies behave after failure in rings of 5 hops; particularly, the worst case for spanning tree protocols is shown, namely when the root bridge fails; box plots include the interquartile range, sample median, extreme measurements and outliers (note logarithmic scaling of *y*-axis). This test is consistent with the IEC 62439-1 Standard [6] and its results corroborate that STP convergence time is considerably slower than RSTP. Moreover, these spanning tree protocols depend highly on the number of nodes and the failure location in the ring, which is limited to 40 hops according to the IEEE 802.1D, although it is true that there are available other layer 2 protocols more suitable for this type of topology, which is detailed in [13]. The size limitation does not occur in OpenFlow networks.

### 5.3. Robustness of the Proposal in Multiple Failure Cases

PRP is not only useful for critical applications where data loss is not permitted, but also it may be relevant in situations where the loss rate may be relatively high, such as analyzed in [36], in which the authors utilize PRP with two redundant wireless channels, improving the overall reliability. This may be applied to tolerate arbitrary faults, such as accidents, natural disasters, malicious attacks or blackouts.

**Figure 6:**
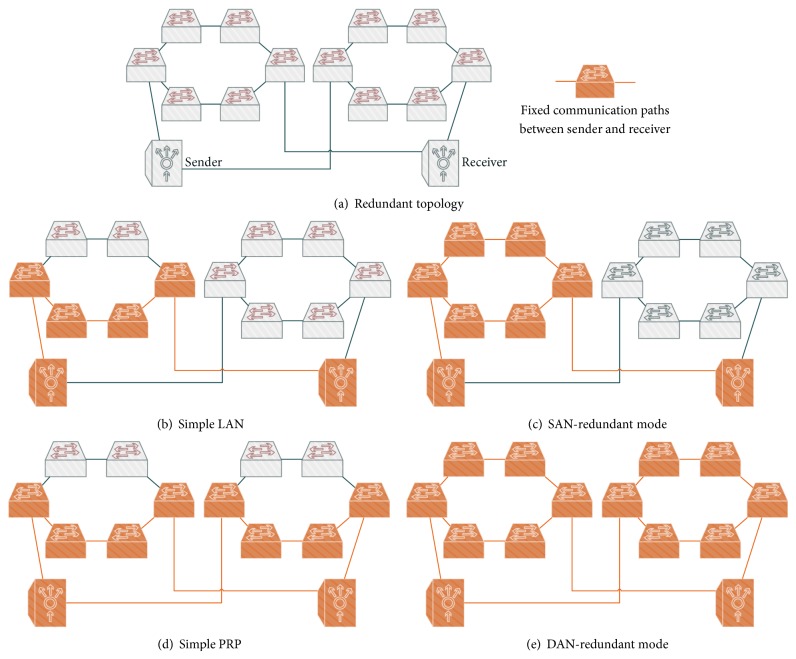
Test cases.

Below, we study different lossy topologies and study scenarios in order to understand the robustness provided by the establishment of multiple active paths. As a test of concept, we generated two identical and independent topologies to which the PRP nodes are connected. Furthermore, both topologies enable the creation of disjoint paths of equal cost, which reduces the possible number of cases and makes the understanding of them easier (Figure 6(a) sketches how this redundancy is organized). We perform a comparative evaluation in terms of recovery time for the following cases:

- simple LAN where only one path is established, Figure 6(b),
- SAN-based operation mode, Figure 6(c),
- simple and common PRP deployment, Figure 6(d),
- DAN-based operation mode with multiple redundancy in both LANs, Figure 6(e).

Regarding the implementation of redundancy, the Static Flow Pusher Floodlight module (Figure 3) is used to set static rules that form parallel paths. As an outcome, Table 1 show the mean global loss rate, taking into consideration the same packet loss rate per link, which is inversely related to their availability, as also considered in [36]. These results show the reduction of global loss rate is achieved by using multiple parallel links. Therefore, our scheme allows to implement different topologies with extremely low packet losses.

## 6. Related and Future Work

Regarding related work, it should be noted that it is the first time that PRP features are included in OpenFlow networks. In comparison with other possible approaches such as [7], which studies the combination of PRP and TRILL networks, they do not provide greater redundancy than conventional PRP deployments and, as asserted by the authors, there are situations where their “proposal cannot cope with the most stringent requirements. Accordingly, this paper encourages practitioners to supplement it by taking into account some principles of the PRP protocol.”

With respect to other layer 2 technologies, we have reviewed standards protocols that have been proposed by industry organizations such as the IEEE or IEC, without considering proprietary solutions or protocols with a limited application field, such as the ARINC 664-p7 or SAE AS6802 specifications. Both of them propose extensions to Ethernet for providing QoS and redundancy mechanisms. Reference [37] discusses the capabilities of both solutions, along with the IEEE AVB (Audio Video Bridging) protocol. In this regard, it is necessary to note the design proposal of the new generation of AVB, through the Time-Sensitive Networking Task Group, that includes IEEE P802.1CB: a recent “Project Authorization Request” to address the development of active redundancy [20].

Concerning future work, nodes implement PRP without changes in the SAN-based operation mode. However, it is clear that the protocol may be modified to reduce its overload and complexity: certain fields, for example, LAN ID, may not be necessary in this configuration mode. Otherwise, the work here presented manages unicast traffic redundancy; thus, we consider it necessary to study how the OpenFlow protocol may provide multicast traffic filtering along active redundant paths.

Furthermore, the platform works as a NOS where new capabilities can be incorporated, but always with a greater focus of meeting manageability and flexibility in the prevention, detection and response to abnormal situations, such as failures or disasters.

## 7. Conclusion

Mission-critical networking applications require redundant topologies that avoid single points of failure for improving the overall network availability. Thus, the establishment of parallel paths affects the network downtime, improving network resilience.

In particular, this paper focuses on PRP, one of the most representative standards for ensuring zero switchover time when a link or switch fails; in which, the redundancy control is responsibility of end-nodes. For the purpose of increasing the availability, this paper shows the importance of maintain multiple redundant infrastructures using OpenFlow. Our solution establishes multiple redundant paths in individual LANs, achieving a minimal disruption in case of multiple failures. Moreover, this proposal enables flexible topologies that are not possible to implement in current deployments.

Additionally, we have presented certain features that facilitate the dynamic resource management according to the network status, making the most of available resources. This shows the benefits of using a centralized external agent that provides adaptability for different services. Hence, our proposal achieves a more flexible network control, fulfilling stringent real-time requirements.

## Figures and Tables

**Table 1:** Comparison of the packet-loss robustness in different topologies.

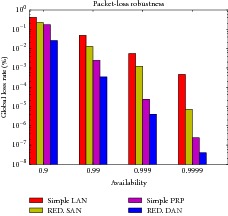				
Availability	Global loss rate (%)			
	Simple LAN	RED. SAN	Simple PRP	RED. DAN
0.9	4.2 × 10^−1^	2.2 × 10^−1^	1.7 × 10^−1^	4.6 × 10^−2^
0.99	4.9 × 10^−2^	1.3 × 10^−2^	2.5 × 10^−3^	3.4 × 10^−4^
0.999	5.5 × 10^−3^	1.2 × 10^−3^	2.4 × 10^−5^	4.0 × 10^−6^
0.9999	4.6 × 10^−4^	7.1 × 10^−6^	2.4 × 10^−7^	4.0 × 10^−8^
